# GANBISS: a new GPU accelerated N-body code for binary star systems

**DOI:** 10.1007/s10569-023-10147-2

**Published:** 2023-05-25

**Authors:** Maximilian Zimmermann, Elke Pilat-Lohinger

**Affiliations:** Department of Astrophysics, University of Vienna, Türkenschanzstraße 17, Vienna, 1180 Austria

**Keywords:** Celestial mechanics—methods, Numerical—planets and satellites, Formation—stars, Planetary systems—stars, Binary stars

## Abstract

We present a GPU accelerated N-body integrator using the Bulirsch–Stoer method, called GANBISS (GPU accelerated n-body code for binary star systems). It is designed to simulate the dynamical evolution of planetesimal disks in binary star systems which contain some thousand disk objects. However, it can also be used for studies of non-interacting massless bodies where up to 50 million objects can be studied in a simulation. GANBISS shows the energy and angular momentum conservation behavior of non-symplectic integration methods. The code is written in CUDA C and can be run on NVIDIA GPUs of compute capability of at least 3.5. A comparison of GPU and CPU computations indicates a speed-up of the GPU performance of up to 100 times—depending on the number of disk objects.

## Introduction

Most of the stars in the solar neighborhood are part of binary or multiple star systems, respectively (up to $90\ \%$ of the O, B-type stars (Moe and Di Stefano 2016), about $50\ \%$ of the solar-type stars (Raghavan et al. 2010) and $27\ \%$ of the low mass stars (Delfosse et al. 2004)). From the detected exoplanet candidates ($\sim 5000$)^1^ only a small fraction ($\sim 217$)^2^ of the planets are located in binary star systems. However, both observations and simulations suggest that planet formation should be possible in stellar systems (e.g., Müller and Kley (2012) or Haghighipour and Raymond (2007)).

While early phases of planet formation have to be studied by hydrodynamical simulations, the late stage of terrestrial planet formation can be investigated using N-body simulations which are less time-consuming. Nevertheless, N-body computations can result in a high computational effort in case of a large number of bodies, as it scales with $N^2$. To greatly increase the performance of such simulations and reduce the time needed for a single simulation one can make use of the parallelization possibilities of graphical processing units (GPU). The first GPU code with an application to planet formation is the GENGA code, which is in its current state able to simulate up to $\sim 60000$ interacting bodies (Grimm et al. 2022). But simulations using GENGA are limited to single star systems due to the chosen symplectic integration method, which are in principal more powerful than classical ones (e.g.,  Runge–Kutta or Bulirsch–Stoer). Symplectic integration methods are especially designed for Hamiltonian systems, so that the conservation of energy is given for a constant step size. Simulations of planetesimal/embryo disks in circumstellar motion of tight binary stars lead to many collisions of the disk objects. Thus, the application of a symplectic integrator would be disadvantageous, since numerical studies of collisions require a variable step size. To overcome this problem existing hybrid codes could be used (e.g.,  Mercury6 (Chambers and Wetherill 1998) or SyMBA (Duncan et al. 1998)) but they are not designed for dynamical studies in binary stars. Therefore, we developed a GPU code using a classical integration method (Bulirsch–Stoer method).

In this study, we introduce the new N-body code GANBISS (GPU Accelerated N-body code for binary star systems), which uses the acceleration by graphical processing units (GPUs). GANBISS can be used (i) for studies of some thousand (up 10000) interacting objects moving in binary stars and (ii) for investigations of some million (up to 50 million) non-interacting massless bodies single or binary stars.

The method and implementation are described in Sect. 2. Section 3 gives a short summary about the two computation modes. In Sect. 4, an overview about the conservation probabilities and a comparison of the performance is given. Finally, a summary and an outlook for future improvements are provided in Sect. 5.

## Method

The force acting on a body $m_\nu$ in an N-body system can be written as:

1 $$\begin{aligned} \varvec{F}_\nu = m_\nu \ddot{\varvec{r}}_\nu = k^2\ m_\nu \sum ^N_{\mu =0, \mu \ne \nu }\frac{m_\mu }{\Vert \varvec{r}_\mu -\varvec{r}_\nu \Vert ^3}\left( \varvec{r}_\mu -\varvec{r}_\nu \right) \end{aligned}$$

where *k* is the Gaussian Gravitational constant. The 3*N* second-order differential equations (1) can be rewritten into 6*N* first-order differential equations

2 $$\begin{aligned} \dot{\varvec{r}}_\nu= & {} \varvec{v}_\nu \end{aligned}$$

3 $$\begin{aligned} \dot{\varvec{v}}_\nu= & {} k^2 \sum ^N_{\mu =1,\nu \ne \mu } \frac{m_\mu \left( \varvec{r}_\mu - \varvec{r}_\nu \right) }{\left\| \varvec{r}_\mu - \varvec{r}_\nu \right\| ^3} \end{aligned}$$

where *n* denotes the current phase space vector for the $\nu th$ particle at time *t* and with the evolution function *f* Eqs. (2) and (3) can be rewritten as

4 $$\begin{aligned} \varvec{y}_{n,\nu }= & {} \left( \begin{array}{c} \varvec{r}_{n,\nu }\\ \varvec{v}_{n,\nu }\end{array}\right) \end{aligned}$$

5 $$\begin{aligned} \dot{\varvec{y}}_{n,\nu }= & {} \varvec{f}\left( t_n,\varvec{y}_{n,\nu }\right) \end{aligned}$$

To solve Eq. 5 we use the Bulirsch–Stoer (BS) method which is a well-known algorithm for integrating the N-body problem. This method is released in several publications such as in the Numerical recipes (Press et al. 2002),Mercury6 (Chambers and Wetherill 1998) or nie-package (Eggl and Dvorak 2010). Basically the method consists of two parts:

(i) The so-called modified midpoint method is used for the integration of time-step $\tau$, which is performed several times using an increasing number of sub-steps $\tau _m=\frac{\tau }{m}$ where the splitting procedure proposed by Deuflhard (Deuflhard 1983) is used:
  $$\begin{aligned} n_m&=1\qquad m=0,\\ n_m&=2\cdot m\qquad m \in \mathbb {N} \end{aligned}$$
(ii) A polynomial extrapolation is applied to each result $R_m$ to obtain the result $R_\infty$. A BS step is successful when
  6 $$\begin{aligned} \varvec{|R_{m-1} - R_{m}|= \epsilon _i < \epsilon } \end{aligned}$$

where $\epsilon _i$ is the error estimate for the column *i* of the extrapolation scheme and $\epsilon$ is the chosen accuracy. Depending on the number of iterations (*i*) needed, the time-step $\tau$ of the next integration step is chosen accordingly to the following scheme:

7 $$\begin{aligned} \left. \begin{array}{ll} \epsilon _i< \epsilon ,\; m< m_\textrm{max} &{} \tau \cdot 1.3\\ \epsilon _i < \epsilon ,\; m = m_\textrm{max} &{} \tau \cdot 0.55\\ \epsilon _i > \epsilon ,\; m\ge m_\textrm{max} &{} \tau \cdot 0.5 \\ \end{array} \right\} = \tau _\textrm{new} \end{aligned}$$

The last case only occurs if the accuracy couldn’t be achieved within the maximum number of iterations $n_\textrm{max}$. The time-step $\tau$ is then halved and recalculated.

### Collision handling

The dynamical evolution of planetesimal disks shows close encounters among planetesimals and planetary embryos. If the distance between two bodies is smaller than their summed radii a collision occurs. However, for the sake of reducing the simulation time a slightly increased collision radius has been used, which is $5\%$ of the Hill radii $r_H$ of the colliding bodies:

8 $$\begin{aligned} d < 0.05 \cdot \left( r_{H,\nu } + r_{H,\mu }\right) \end{aligned}$$

In case the colliding bodies orbit a single star their Hill radii are:

9 $$\begin{aligned} r_H \approx a \cdot \left( \frac{m}{3M}\right) ^{1/3} \end{aligned}$$

where *a* is the semi-major axis of the disk object, *m* its mass and *M* the mass of the star.

In case the colliding bodies orbit both stars (i.e.,  P-type motion), their Hill radii are:

10 $$\begin{aligned} r_H \approx a_\textrm{bary} \cdot \left( \frac{m}{3\cdot \left( M_1 + M_2\right) }\right) ^{1/3} \end{aligned}$$

where $a_\textrm{bary}$ is the distance of the disk object to the barycenter of the two stars, *m* is the mass of the disk object and $M_1$ and $M_2$ are the masses of the two stars. For simplicity the collision is handled as a perfect inelastic collision, such that the two bodies (with masses $m_1$ and $m_2$) merge to a bigger one ($m_\textrm{coll} = m_1 + m_2$). The new positions and velocities of the resulting body correspond to those the center of mass of the two original bodies.

### GPU-implementation in CUDA

The principal idea of GPU computing is the parallel computation on thousands of cores using the SIMT execution model (single instruction, multiple threads). As the name implies, a single instruction is applied on a given number of threads, where a thread is a single execution sequence. Thirty-two threads are combined to a so-called warp. Each thread within a warp executes the same instructions. On the logical scale, threads are combined up to three dimensional thread blocks, which are themselves combined to two dimensional grids. The GPU functions (kernels) are executed on a given grid.

There are different memory spaces with different access latencies which can be accessed by the threads. The lowest latency have the registers, which can only be accessed by the thread its belonging to. Each thread block has shared memory, which can be accessed of all threads of the block. The slowest access occurs on the global memory, but every thread of each block has access to it.

There are differences between a GPU and a CPU. So, not the complete code is transferred on the GPU, only parts which benefit from the parallelization of their task. A GPU has a higher overhead time in their functions (kernels) calls and a lower clock rate compared to a CPU. To hide this additional work the GPU need some degree of parallelization of their computation. One has also to take into account that the instruction set of a GPU is limited to that of a CPU. These instruction sets are optimized for floating-point and arithmetic calculations, making a GPU very efficient in computational tasks which allow a high degree of parallelism. In practice the code gets split into a host part (executes on the CPU) and a device part (executes on the GPU). Where the device code is called from the host and includes all kernel functions. For detailed description of the CUDA model and interface see the CUDA programming guide^3^.

In the next section, the parallelized parts of the codes are discussed.

#### Overview of the kernels

As the N-body problem scales with $\tau \sim \textrm{O}\left( N^2\right)$ due to the right-hand side of the equation of motion (3), it is the most crucial part to parallelize. Thus, the following kernels have been parallelized:

- *bb_force*: computes the $N^2$ forces
- *update_arr*: corresponds to all the computations of the sub-steps of the BS method.
- *extrapol*: is for the recursive extrapolation.
- *errmax*: finds the largest error among all computed bodies using a parallelized reduction algorithm.
- *det_merg_ev*: checks for collision events by computing all-pair distances as it is performed in the *bb_force* kernel.

#### Simple kernels: update_arr

The *update_arr* refers to the simple kernels which do not have any dependencies between the bodies. Thus, it is simple to parallelize with each thread deal with one body. These kernels are the update steps of the modified midpoint method, the update of the extrapolation scheme and the check for the crossing of the cutoff radius, as well as the extrapolation. Yet, the effort of the extrapolation is depending on the number of sub-steps *m*.

#### Force computation: bb_force

**Fig. 1:**
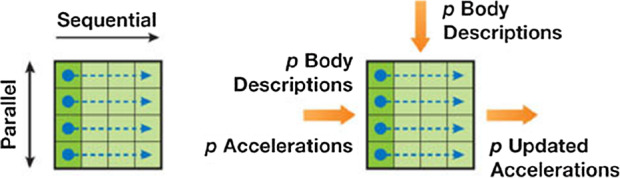
Schematic figure of the computational tile. The left panel shows the separation into the parallel and sequential part. The right panel the inputs needed for the calculation of $p^2$ interactions and the *p* results gained. The figure is taken from Nyland et al. (2009)

Equation 3 is an all-pair algorithm, because each entry $f_{\nu \mu }$ of a $N\times N$ grid has to be computed. The total force $f_\nu$ acting on one particle $\nu$ is the sum of all entries within the $\nu$-th row. The computation is split into a parallel and sequential part (Fig. 1). Therefore the grid is partitioned into squared computational tiles, each performing a parallel and sequential part. A tile consists of $p_\nu$ rows and $p_\mu$ columns, which are stored in registers ($p_\nu$) and shared memory ($p_\mu$). After each sequential pass of a computational tile the bodies acceleration $a_\nu$ gets updated.

In practice, these tiles are represented by thread blocks. Thus, the size of a tile is equal the number of threads per block squared. Each thread $p_\nu$ computes $p_\mu$ interactions sequentially and updates the acceleration $a_\nu$ at the end of the tile. After $p^2$ interactions a synchronization is performed, the next $p_\mu$ body descriptions are loaded into the shared memory and are computed. This is repeated until all $\mu$ interactions per body have been evaluated. One exception is the last tile. Because the number of bodies varies and are not a multiply of the number of threads at the complete simulation time, the last tile has a reduced number of $p_\mu$ computations. The same is true for the last parallel tile with a reduced number of $p_\nu$ threads.

**Fig. 2:**
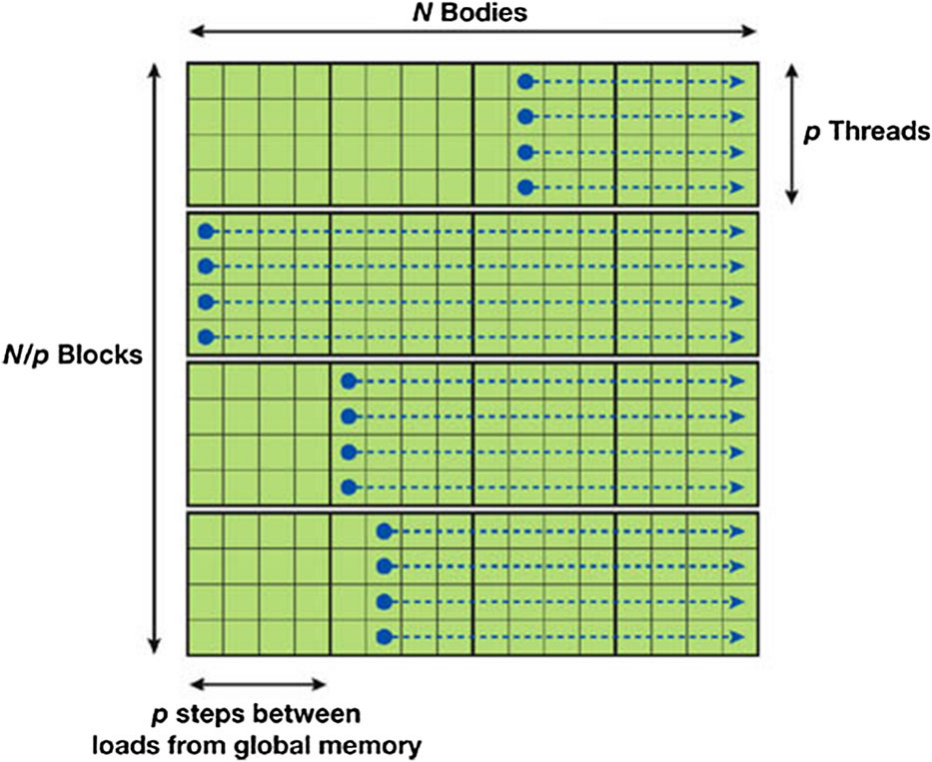
The full scheme of the computation of all pair-wise interactions for the example case of $N=16$ and $p=4$. This figure is also taken from Nyland et al. (2009)

For a simulation with *N* bodies and *p* threads per block there are *N*/*p* tiles for $N\mod p=0$ and $N/p+1$ tiles for $N\mod p\ne 0$. Figure 2 shows a full example grid for $N=16$ and $p=4$. For a more detailed description of the algorithm, see the work of Nyland Nyland et al. (2009).

This algorithm reduces the memory load because of the reuse of the data stored in the shared memory as well as a full utilization of the GPU.

#### Force computation massless: bb_force_ml

The computation of $f_{\nu \mu }$ for the massless particles works nearly the same way as in the $N^2$ case. Yet, the exception is the input of the computational tile changes. $p_\nu$ corresponds to the massless particles and $p_\mu$ to the massive bodies acting on the massless particles. The computational effort for the massless particles reduces to $N\cdot M$, where *N* is the number of massive bodies and *M* the number of massless bodies. Depending on the number of threads per block and the number of the massive bodies full utilization of the GPU may not be achieved anymore.

#### Determination of $\epsilon _\textrm{max}$: errmax

**Fig. 3:**
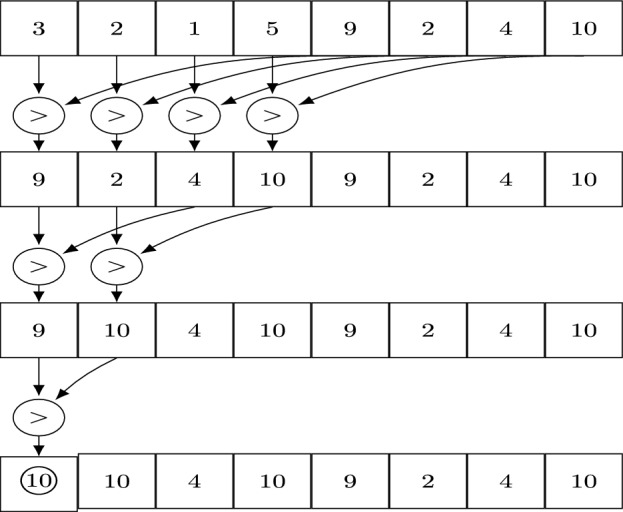
A schematic view of the reduction procedure for an array size of 8. In this example the largest integer is searched. The algorithm requires an array size of $2^n,\quad n>0$

The search for the largest error has to be performed with a parallel reduction. First a sample of the largest errors of the position and velocity vectors of all bodies will be determined with the size of the block size. This is achieved by iterating through all bodies in parallel, where each thread within the block is assigned a body $p_{\textrm{threadId} + \textrm{blockSize} * i}$ with *i* being the loop index. The computed errors will be compared with the errors from the previous loop index. In the next step the reduction on the remaining sample will be performed. Therefor the error values are stored in the shared memory. The values with $j<ArrSize/2$ will be compared with $j+ArrSize/2$. The results are stored at their positions and cut the number of the remaining values into half. This is procedure is repeated until the highest error remains. Figure 3 shows this reduction procedure for an array size of $ArrSize=8$.

As in the simulations the number of bodies may vary because of collisions and/or ejections, the arrays may not be filled completely though these are filled with zeros before each kernel execution.

#### Detection of collisions: det_merg_ev

The detection of collisions works similar as the force evaluation. Yet, it is an all-pair comparison. Instead of the computation of the accelerations the kernel checks if the distance of two bodies is smaller than the given radii following the inequality 8. If this inequality holds a collision flag is set and the index of the two colliding bodies are stored. The collision itself is performed in a CPU function.

The ***det_merg_ev*** kernel is a separate function, because the additional query of the collision condition in the ***bb_force*** kernel would lead to a divergence in the parallelization and thus to a longer calculation time in the ***bb_force*** kernel.

## Applications

### Default-mode

By default, an N-body simulation take the interactions of all bodies (stars, planets and disk objects—i.e.,  planetary embryos and planetesimals) into account. The simulations can be carried out for single or binary stars. For a binary star system S- or P-type motion has to be distinguished. In case of S-type motion, the disk is set around the primary star and in the case of P-type motion, the disk is around both stars with respect to binaries barycenter. The disk objects are allowed to collide with each other. Collisions between stars and planets are not explicitly treated, since such collisions are not expected in the intended simulations, or it is presupposed that planets, especially gas giants, within the simulation are on stable orbits. Moreover, disk objects can be ejected from the system. The cutoff radius, which defines the distance a body has to reach to be ejected from the system can be set by the user. In addition, the simulation time, the output time-steps and the accuracy of the BS integration ($\epsilon$) are defined by the user.

### Massless particles

Massless particles have been introduced to simulate a large number of bodies where their mutual interactions are negligible. Thus, they do not have any gravitational influence and they can only collide with massive objects (stars or planets)—but in such a case they will be removed from the simulation. The computational effort for the massless bodies reduces to $\tau \left( N\cdot M\right)$ where *N* corresponds to the number of massive bodies and *M* to the massless bodies.

GANBISS has been successfully applied to the study of comets in the Oort cloud perturbed by a passing star (see (Pilat-Lohinger et al. 2022), Loibnegger et al. (2022)).

## Results

### Conservation of angular momentum and energy

**Fig. 4:**
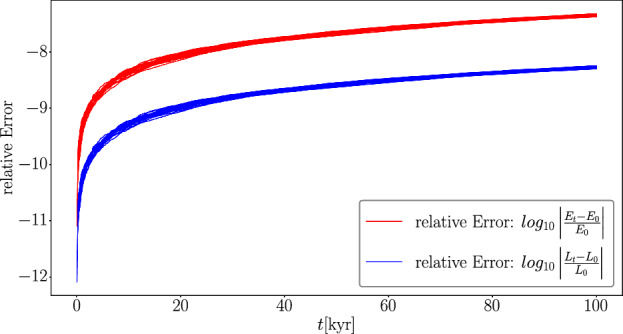
The relative error of the total energy (red lines) and total angular momentum (blue lines) in a logarithmic scale. The figures contain all 20 setups and are simulated for $100\ \textrm{kyr}$

To check the reliability of the presented code, its conservation of the total energy and the total angular momentum within a given system has been tested. For non-symplectic integrators the momentary relative error in the energy and angular momentum follows roughly a linear growth. Figure 4 shows the momentary relative error of the energy and angular momentum for 20 different simulations for $100\ \textrm{kyr}$ simulation time. Each simulation consists of a solar mass central star and 17 planetary embryos distributed differently between 1 and $5\ \textrm{au}$ around the central star. All simulations show the expected error behavior for non-symplectic integrators. The relative error itself is quite robust among the different initial configurations.

Collisions performed via perfect merging do not conserve energy. Thus, due to the choice of the integration method, one can only try to keep the error of the energy within a certain error range.

Note, that the “loss” of energy through collisions and ejections is not considered. Thus, the energy may not preserved in simulations with collisions or ejections.

### Performance

Performance measurements have been taken using the NVIDIA profiling tools^4^ to obtain each kernel’s performance. For the comparison with the CPU Fortran code the *gprof* profiling tool has been used. The initial set-ups for the comparisons consist of a binary star system with a planetesimal disk in S-type motion. Both stars are Solar mass stars and have a separation of $a_b=30\ \textrm{au}$. The planetesimal disk is placed around the primary star between 1 and $4\ \textrm{au}$ with varying number (10-10000) of small bodies and a total mass of about $M_\textrm{tot}\approx 2.4 M_\oplus$.

#### Performance of the main kernels

**Fig. 5:**
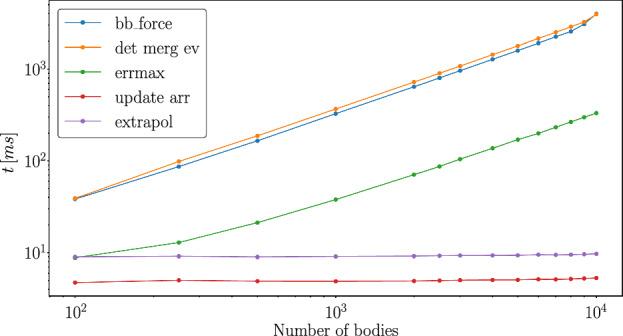
Average kernel evaluation time for different numbers of bodies on a NVIDIA A100

Figure 5 shows the average computation time for the main kernels as a function of the number of bodies.

The most expensive kernels are the force evaluation (blue line) and the collision detection (orange line) in Fig. 5), because both kernels perform all pair-wise interactions. While the *update_arr* (red line) and the *extrapol* (purple) referring to the simple kernels are the least expensive kernels as they have only a *N* dependency. In between is the *errmax* (green line) kernel. This kernel’s performance is limited by the block size as the reduction algorithm is performed within block.

**Fig. 6:**
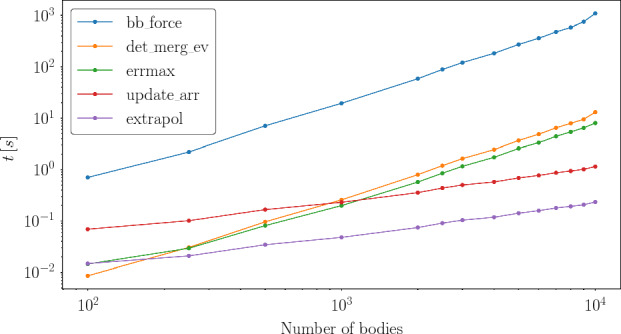
The total time needed of each kernel for a simulation time of $10\ \textrm{yr}$ considering different numbers of bodies on a NVIDIA A100

Comparing the total time needed by the kernels within a simulation run, the time effort changes (Fig. 6), because of the number of calls of each kernel. While the *det_merg_ev* kernel is called once per successful time-step, the kernels *errmax* and *extrapol* are called once per iteration step within a time-step and the kernels *bb_force* and *update_arr* are called several times per iteration step. The small decline from 9000 to 10000 objects is due to a smaller number of function calls. The *bb_force* kernel is the most time-consuming one during the simulation runs and needs up to $99\ \%$ of the computation time.

#### Performance comparison between GPU and CPU

**Fig. 7:**
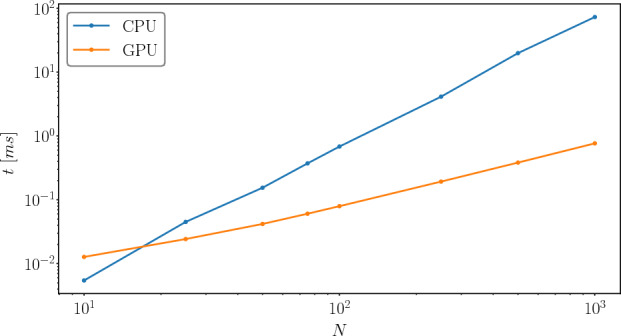
Average computation time for the force calculation kernels for a CPU (Intel Core i7-8700T @ 2.4GHz) and the here presented GPU (AMD EPYC 7713 @ 2.0GHz, NVIDIA A100) implementation

Figure 7 shows a comparison of the force computation between a CPU (nie-package (Eggl and Dvorak 2010), Fortran implementation) and the here presented GPU implementation. The simulations have been carried out with $10 - 1000$ planetesimals with a total planetesimal mass of $2.4\ M_\oplus$. The GPU implementation is up to 100 times faster compared to the CPU implementation depending on the number of bodies. However, for a small number of bodies ($<20$) the CPU code is faster. This is because the GPU cannot benefit fully from the parallelization and is limited by the kernel launch overhead. Despite the number of cores there are other differences between a CPU and a GPU. A CPU has lower latency per instruction and a higher clock rate, which favors the performance for a small number of bodies.

#### Performance comparison between different GPUs

**Fig. 8:**
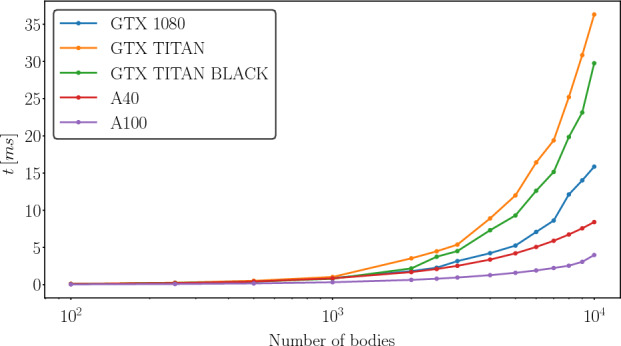
Comparison of the average time needed of the *bb_force* kernel for different GPUs for up to 10000 bodies

Figure 8 shows the performance for different GPUs for the force computation. The A100 shows the best results because it has the best theoretical double precision performance. For relatively small numbers ($\sim 1000$ bodies) the computation time of the force barely differs between the different GPU models. Hence, all used GPUs are suitable for the computation of small numbers of bodies. For larger numbers of bodies the computation times diverge visibly and for more than $>5000$ bodies only two GPUs (A100 and A40) are recommendable.

While the GTX TITAN cards should be the second best cards on paper according to the theoretical floating point operation performance, they perform the worst in this comparison. This is probably because the calculation of the theoretical performance of the floating point operations do not consider the clock rate or the memory bandwidth, which is faster in case of the GTX 1080 and A40.

### Performance massless

**Fig. 9:**
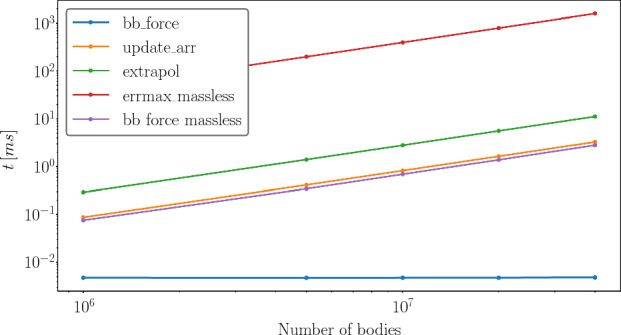
The average computation time for different kernels for different numbers of massless particles on a A100

Figure 9 shows the average computation time for the main kernels for simulations with massless particles. In these simulations $10^6$ to $4\cdot 10^7$ massless test-particles have been placed around a star between 50 and $5000\ \textrm{au}$ to mimic the outer Kuiper belt region and the disk of the inner Oort cloud. In addition a gas giant orbits the star at $5.2\ \textrm{au}$. The time needed for the force evaluation of the massless particles shows an *N* dependency and thus, is not the dominating factor in the computation time anymore. The bottleneck now is the calculation of the maximum error. This is because the performance is limited by the block size, which is 1024 threads per block. For two massive bodies the time needed for the computation of their interactions is neglectable.

### Example: planetesimal disk in binary star systems

A simulation of a self-gravitating planetesimal disk in a binary star systems has been carried out for $1\ \textrm{Myr}$ simulation time. The binary stars have a separation of $30\ \textrm{au}$ and move on circular orbits in the same plane. Both stars have a mass of $1\ \mathrm {M_\odot }$. The disk contains 2000 planetesimals and 25 planetary embryos (Moon to Mars sized) with a total disk mass $M_\textrm{disk} \approx 4.8\ M_\oplus$, where the total mass of planetesimals and embryos is equal. We assume the existence of planetesimals in a circumstellar disk of tight binary stars due to the work of Gyergyovits Gyergyovits et al. (2014). All disk objects are placed around the primary star between 1 and $4\ \textrm{au}$ and are initially dynamically cold.

**Fig. 10:**
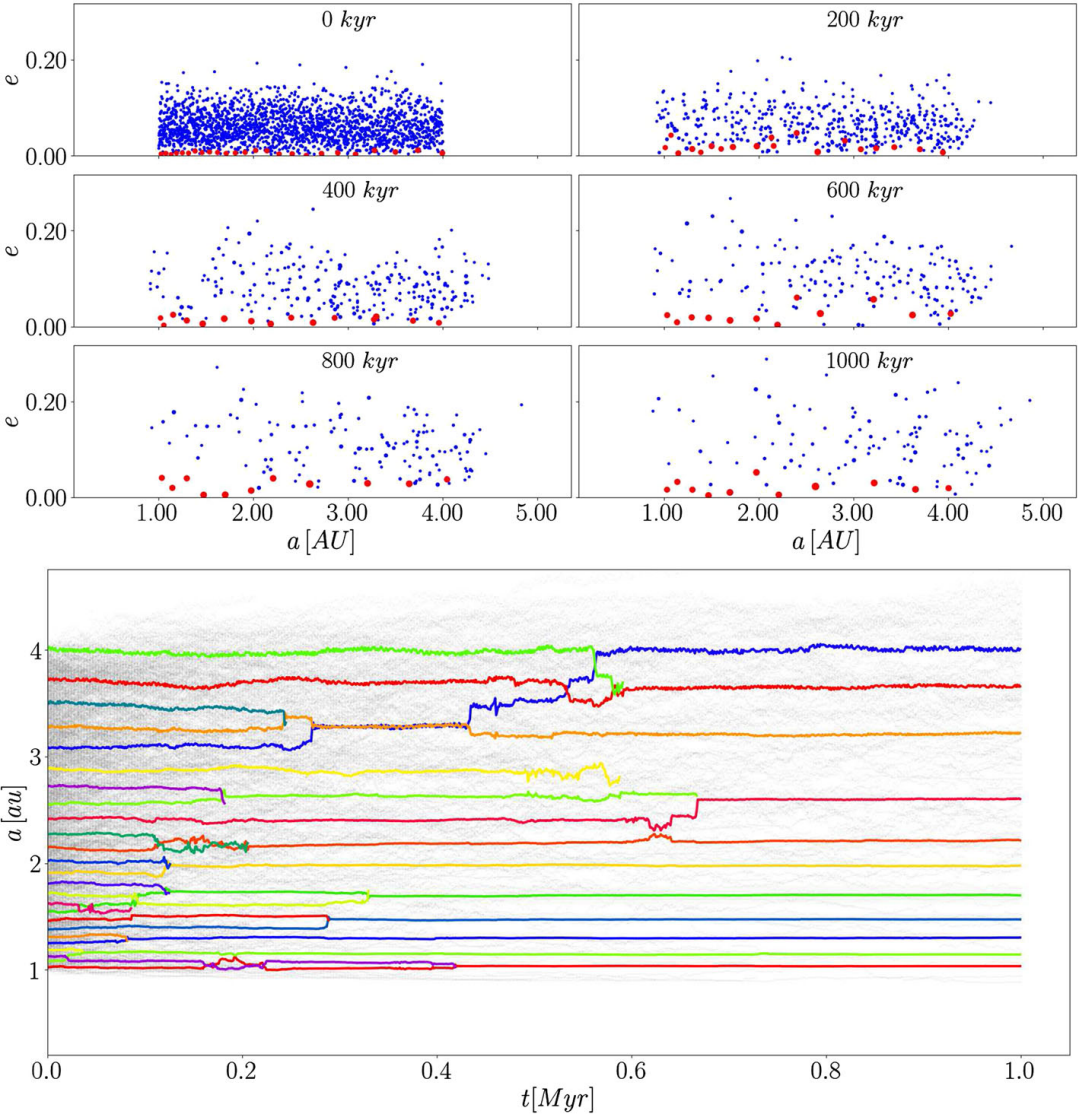
The upper six panels show the disk objects semi-major axes and the eccentricity for six different snapshots within the $1\ \textrm{Myr}$ simulation time. The blue dots represent the planetesimals and the red dots the planetary embryos. The bottom panel shows the evolution of the disk objects semi-major axis for the whole simulation time. Different colors indicate the various planetary embryos and the light grey lines show the planetesimals. Collisions between embryos are clearly seen and the greyscale indicates the decreasing number of planetesimals due to the collisions with embryos

Figure 10 shows 6 snapshots of the planetesimal (blue dots)/embryo (red points) disk for the computation time of $1\ \textrm{Myrs}$. The top panels indicate clearly that most of the planetesimal collided with embryos within the first $200\ \textrm{kyrs}$. Occasionally mutual embryo collisions occur (see Fig. 10 bottom panel). Due to numerous collisions the circumstellar disk is reduced from 25 embryos to 11 and from 2000 planetesimals to 102 after $1\ \textrm{Myrs}$. Besides the mutual interactions of planetesimals and embryos only perturbations of the secondary star act on the disk since no giant planet has been included in the initial configuration. An additional giant planet would lead to a higher number of collisions and an increased impact velocity (Thébault et al. 2004).

## Conclusion and Outlook

In this study, we introduced a new GPU parallelized N-body code so study circumstellar disks in binary star systems. A performance analysis has been presented which indicates the efficiency of the GPU Code especially for a high number of interacting bodies where a speed-up of the computation up to 100 times of the CPU performance has been achieved. For a small number of bodies ($<20$) the GPU code is slower compared to the CPU code.

In addition, it has been shown that GANBISS can be applied to study the dynamical behavior of massless bodies where the computational effort is smaller compared to a fully interacting system of some thousand bodies.

As an example, we showed the growth of planetary embryos in a circumstellar disk in a binary star via collisions which were studied in a first attempt by perfect merging. This simple consideration of collisions will be improved in future studies by including results of SPH^5^-simulations which enable a detailed analysis of two body collisions and provide more realistic results.

Another problem is the $N^2$ complexity of the N-body problem. While a GPU implementation increases the performance due to its increased arithmetic power, it depends strongly on the underlying hardware. This problem will be additionally addressed in future work by implementing a method that reduces the all-pair interactions to body-cell or cell–cell interactions. These methods are known as Barnes-Hut (Barnes and Hut 1986), or fast multipole method (Greengard and Rokhlin 1987) in the latter case.

Currently, GANBISS is still in an alpha version, but it is planned to make it publicly available in the future.

## Data Availability

The datasets generated during and analyzed during the current study are available from the corresponding author on reasonable request.

## Footnotes

1 https://exoplanet.eu.

2 https://adg.univie.ac.at/schwarz/multiple.html.

3 https://docs.nvidia.com/cuda/cuda-c-programming-guide/index.html.

4 https://docs.nvidia.com/cuda/profiler-users-guide/index.html.

5 Smooth particle hydrodynamics.
